# Prescription Renewal Request Reviews by Student Pharmacists in a Family Medicine Clinic

**DOI:** 10.3390/pharmacy9040197

**Published:** 2021-12-12

**Authors:** Jarred Prudencio, Michelle Kim

**Affiliations:** The Daniel K. Inouye College of Pharmacy, University of Hawaii at Hilo, Hilo, HI 96720, USA; msk@hawaii.edu

**Keywords:** family medicine, primary care, ambulatory care, prescription, renewal, education

## Abstract

Prescription renewal requests were reviewed by student pharmacists on advanced pharmacy practice experiences (APPE) at a primary care and family medicine clinic. Student pharmacists reviewed requests and triaged them to the respective primary care provider (PCP), along with any recommendations to optimize the medication regimen. This study aims to assess the acceptance of these recommendations as well as the student’s perception of this activity as a learning tool. A total of 35 4th-year pharmacy students participated in this activity during APPE rotations from May 2019 to March 2021. A total of 184 recommendations were made, with 128 (70%) being accepted by PCPs. Based on a post-rotation anonymous survey, students reported high levels of agreeance that this activity had a positive impact on their education in a variety of ways. This prescription renewal request review process has been shown to have a positive impact on patient care and clinic workflow while also providing pharmacy students with a helpful educational activity.

## 1. Introduction

The East Hawaii Health Clinic (EHHC) is a primary care and family medicine clinic located in Hilo, Hawaii, with a rural health clinic designation. The EHHC serves as a primary care training site for family medicine residents, pharmacy students, medical students, nursing students, and clinical psychology learners. The clinical pharmacy service at the EHHC primarily focuses on comprehensive medication management, but also includes other functional duties, such as handling prescription renewal requests, prior authorizations, and other medication-related tasks. This service is provided by two clinical pharmacist faculty from the Daniel K. Inouye College of Pharmacy (DKICP) at the University of Hawaii at Hilo.

While many clinics do have clinical pharmacy services, many clinics do not, as having an ambulatory care pharmacist is not a requirement. However, ambulatory care pharmacy is a growing area of pharmacy practice and is becoming more common, as the evidence supporting the integration of a clinical pharmacist in the outpatient clinic setting is growing. While many clinics do have pharmacy services, the responsibilities and logistical operations of the pharmacy services can vary greatly.

Since the EHHC is a primary care clinic, a large part of the clinic’s responsibilities is to manage patients’ chronic conditions in a comprehensive manner. One aspect of this patient care is through medication management. When prescribing a medication, a provider will write a prescription order, which will include a quantity and number of refills. Once the number of refills on a prescription are used, a prescription renewal is needed to have the patient continue the medication. A prescription renewal is a new prescription order written by a provider for a medication that a patient is already taking. Though the clinic functions in an integrated team approach, the decision on whether to order a prescription renewal is ultimately the responsibility of the patient’s primary care provider (PCP), who, at the EHHC, is a physician or nurse practitioner. There are many techniques and approaches that PCPs use to manage this task, but there is no standardized process for prescription renewal request reviews [1,2]. Since PCPs are tasked with many responsibilities, it can be helpful to have another clinician collaborate in ensuring optimal patient care is provided [3].

A primary responsibility of pharmacists is to ensure medication therapy is safe and effective for patients. This responsibility is carried out by pharmacists across many different settings in different ways, including ambulatory care, hospital, and community pharmacy. DKICP fourth-year pharmacy students on Advanced Pharmacy Practice Experiential (APPE) rotations at the EHHC reviewed prescription renewal requests received via fax from community pharmacies, to ensure optimal medication prescription in a safe and effective manner. 

**Figure 1:**
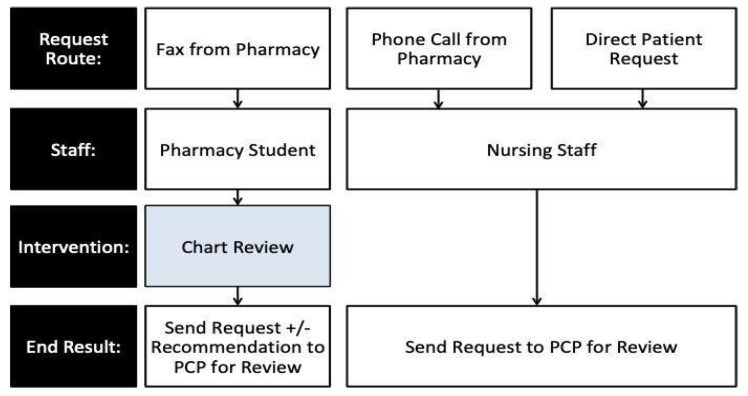
The EHHC Prescription Renewal Request Review Process.

When a patient does not have any refills left on a prescription, a prescription renewal request is sent to EHHC. This can be performed by three main avenues: (1) the patient contacts the EHHC directly, (2) the community pharmacy calls in a verbal request or electronic request to the EHHC, or (3) the community pharmacy sends a faxed request to the EHHC. For options 1 and 2, a nursing staff member or physician at the EHHC receives the request and immediately directs the request to the specific patient’s PCP for review. In option 3, a pharmacy student reviews the faxed request, and then sends the request to the PCP for review. Figure 1 depicts this process. While pharmacy students are on rotation, one of their responsibilities is to review these requests to check for the appropriateness of the prescription. This process was created to provide the pharmacy students with an additional medication-related educational opportunity, while also providing them with an opportunity to contribute to the patient care needs of the clinic. 

Upon receiving a faxed prescription renewal request, the pharmacy student first identifies the patient’s chart in the electronic medical record (EMR). The student then begins to review the prescription for appropriateness and optimization. The students review the prescription request in the context of three main areas: safety, efficacy, and convenience. The specifics of what is reviewed vary widely depending on the medication in question, but the general process is described in this paper. Upon reviewing patient safety concerns, the two primary aspects that should be assessed are laboratory results and drug interactions. For example, if a medication is cleared through the kidneys, it must be ensured that the patient has had a recent metabolic panel with updated renal function status, and that the dose is appropriate, given that renal function. When reviewing for efficacy, things to consider may include laboratory results, vitals, and other chart notes. For example, lipid panel values should be checked if the request is for a lipid-lowering medication, or a blood pressure and heart rate reading would be appropriate to check for the efficacy of an antihypertensive medication. Lastly, checking for convenience includes assessing if the dosage form, quantity, and refills are optimized for the patient. The following is one example of the full process: a request is received for metformin 1000 mg tablets with an original quantity of 60 tablets, and directions to “take 1 tablet by mouth twice daily.” Upon receiving this request, students would identify the patient’s chart, conduct a drug interaction check, and assess the patient’s estimated glomerular filtration rate and liver function test results to ensure this dose of metformin is appropriate to limit the risk of lactic acidosis. Next, students would check to see if the hemoglobin A1c results are current and if it indicated good glycemic control. Lastly, students would check how long the patient has been taking metformin, and if it would be appropriate to increase the quantity and/or refills to allow the patient to have a larger supply, and if the patient has an appropriate follow-up appointment scheduled at the EHHC. When a student identifies a potential intervention or recommendation when reviewing a request, the student presents the case to the pharmacist preceptor to ensure that the recommendation is appropriate. If approved by the preceptor, the student then forwards the prescription renewal request and recommendation to the patient’s PCP. The PCP would then review the renewal request, and after making their own assessment, decide whether to write a new prescription order for the renewal. The PCP may also consider the students recommendation.

This prescription renewal request review process serves the dual purpose of contributing to patient care and as a learning activity for the students. This retrospective study is conducted to assess whether pharmacy students make an impact on patient care through this process and to assess whether it is an effective learning process for the students.

## 2. Materials and Methods

This study was reviewed and approved by the University of Hawaii Institutional Review Board. DKICP pharmacy students from the Class of 2020 and 2021 that completed ambulatory care APPE rotations at the EHHC took part in this activity, from May 2019 through March 2021. Each APPE rotation was 6 weeks in duration and students completed the prescription renewal request review 3 days per week on average. There are two different pharmacist faculty preceptors at the EHHC that precept distinct APPE rotations and do not co-precept. After the completion of a six-week rotation, students were sent an anonymous survey to gather their perspective on this activity as a learning tool. The survey consisted of 7 statements regarding different aspects of this activity, and students responded to each statement via a 5-point Likert scale, ranging from “Strongly Agree” to “Strongly Disagree”. Statements included topics such as the impact of the activity on student’s knowledge of drug monitoring parameters, confidence regarding written interprofessional communication, and confidence in order verification. The survey contents were created by the authors of this study based on educational aspects that were deemed related to the renewal review process.

After all rotations were completed, pharmacists retrospectively reviewed the EMR to identify whether the student’s previous recommendations were accepted. Each recommendation was categorized into the following eight categories: (1) follow-up appointment, (2) order labs, (3) discontinue medication, (4) new medication, (5) therapeutic interchange, (6) quantity change, (7) dose adjustment, and (8) dosage form change. The follow-up appointment category involved recommendations to have the patient scheduled for an in-clinic follow-up appointment based on the review, acknowledging that there were no upcoming appointments scheduled for that patient and it would be clinically relevant to do so based on the medication in question. Recommendations for ordering labs could be based on laboratory monitoring for safety reasons (i.e., renal function, liver function, etc.) or for efficacy monitoring (i.e., thyroid stimulating hormone for levothyroxine). Recommendations for discontinuing a medication were made if the medication in question was deemed inappropriate. A new medication was recommended if a missing standard of care medication was found during the chart review (i.e., a statin for a patient who has a history of stroke). Therapeutic interchange was recommended if either the medication was too high cost for the patient or there was a more appropriate alternative to the medication being requested. Quantity changes included recommendations to change the quantity per fill (i.e., 90 tablets instead of 30 tablets) or the number of refills allowed (i.e., 4 refills instead of 1 refill). Dose adjustments were recommended if the dose was not optimized based on indication, safety, or efficacy. Lastly, dosage form changes were recommendations that included changing the dosage formulation for the medication (i.e., a 40 mg tablet instead of taking two 20 mg tablets, changing from tablets to capsules, etc.). For all categories, recommendation outcomes were noted as either being accepted, declined with a reason provided by PCP, or declined without a provided reason.

Given that the purpose of this activity was dually focused on impact on patient care as well as the pharmacy student’s education, this study aims to assess both. The primary outcome regarding impact on patient care is the number of recommendations made and the percentage of those recommendations that were accepted by the PCP. The impact on pharmacy student’s education is assessed by the survey results. All data presented are analyzed via descriptive statistical analysis. 

## 3. Results

A total of 35 4th-year pharmacy students completed APPE ambulatory care rotations at the EHHC from May 2019–March 2021 and were included in this study. There were 2–3 students on rotation at the EHHC at a time. 

### 3.1. Patient Care Impact: Prescription Recommendations Results 

**Figure 2:**
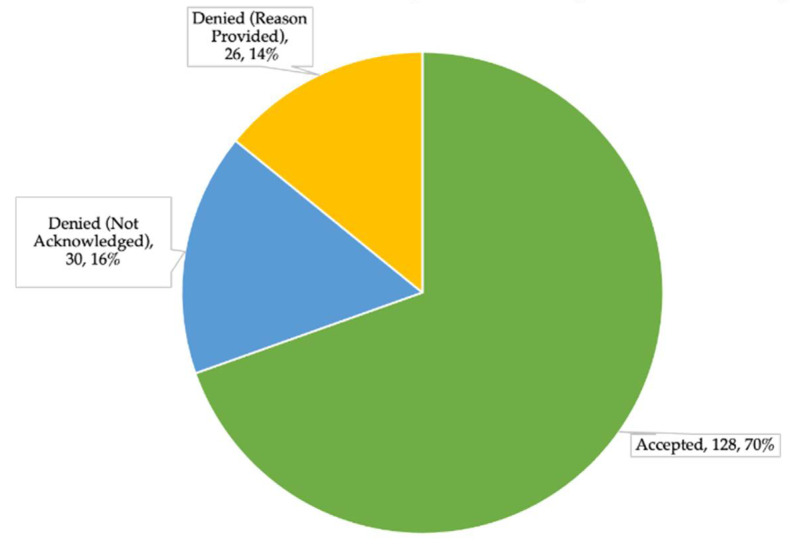
Acceptance Rates of Recommendations Made.

During the study period, a total of 184 recommendations were made by student pharmacists to PCPs via the prescription renewal request review process. Of the 184 recommendations, 128 (69.57%) were accepted and implemented by the PCPs, and 56 (30.43%) were not accepted. Of the 56 that were not accepted, PCPs provided a reason for not accepting the recommendation for 26 of the recommendations, while 30 of the recommendations were not acknowledged. This acceptance rate is depicted in Figure 2.

**Figure 3:**
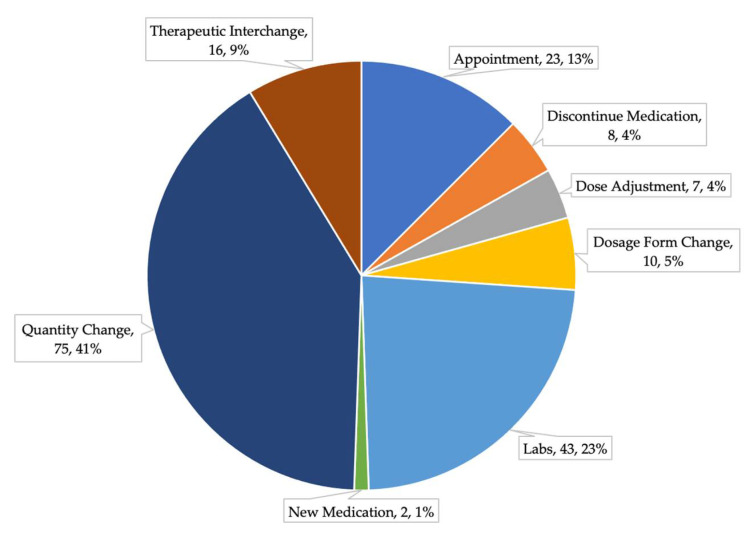
Categories of Recommendations Made.

Of the 184 recommendations made, the most common category was recommendations for quantity changes (75, 40.76%), followed by ordering laboratory tests (43, 23.37%), and scheduling follow-up appointments (23, 12.5%). The least common recommendation category was recommending new medications, with only two recommendations made. Figure 3 depicts the full breakdown of recommendations made by the eight pre-specified categories.

The categories with the highest acceptance rates were therapeutic interchange (81.25%), quantity change (78.67%), and dosage form change (70%). The category with the lowest acceptance rate was recommendations made to adjust doses. Table 1 provides the acceptance rates for each of the eight pre-specified categories.

### 3.2. Educational Impact: Survey Results 

After the completion of the rotation, all 35 student pharmacists completed the anonymous survey (100% response rate). The statement with the highest level of agreeance was that students agreed that the knowledge and skills learned during this process can be applicable to other pharmacy settings in addition to ambulatory care. Overall, there was a high level of agreeance to all seven statements, with > 80% of students selecting either agree or strongly agree for each statement. Table 2 provides the full results of the survey.

## 4. Discussion

The findings from this study demonstrate that this prescription renewal request review process was helpful for patient care. A total of 69.57% of the recommendations made by student pharmacists were accepted and implemented by primary care providers, which can be viewed as a significant impact on patient care. The category of recommendations for quantity changes was the most common recommendation and had the second-highest acceptance rate. While quantity changes may seem simple and may not take a high level of clinical judgement, quantity changes can have a large impact on the patient and clinic workflow. For example, if a prescription is written for a 30-day supply and no refills, but continues to be renewed every month, it means that every month the community pharmacy or patient need to request the medication, and the PCP needs to take the time to review the medical record and send a prescription renewal. With recommendations to increase the prescription to a 90-day supply or adding refills, this decreases the number of times that the patient and community pharmacy need to contact the PCP and decreases the number of times that the PCP needs to review that medical record. The category with the highest acceptance rate was therapeutic interchange. A common example of a therapeutic interchange is the adjustment of a beta-blocker due to receptor subtype specificity (i.e., changing carvedilol to metoprolol to avoid future respiratory exacerbations in a patient taking albuterol). These seemingly small impacts can add up and make the overall process more efficient. The categories with the least number of recommendations and lowest acceptance rates were for discontinuing medications, adjusting doses, and starting new medications. This may be because these recommendations are of a higher level and PCPs may not feel comfortable adjusting the medications without having an appointment with the patient. PCPs review these recommendations without having the patient in front of them, so many may be hesitant to make medication adjustments in this context. For example, a recommendation to increase lisinopril from 10 mg daily to 20 mg daily due to uncontrolled hypertension may not be as easily accepted, as PCPs may not feel comfortable adjusting the dose of the medication without seeing the patient first. It may be possible that PCPs took note of these recommendations and may have used them at future visits, but because of the retrospective nature of this study, that cannot be assessed and it is simply a speculation of possibilities.

Another study that included a control group reported that the prescription renewal request review by a clinical pharmacist improved the patient care process [4]. The study reported that the pharmacist was able to identify significantly more medication-related problems and address medication changes compared to the control group without pharmacist involvement. In other institutions, prescription renewals are a task that is managed solely by ambulatory care pharmacists [5]. Protocols are in place, and if a prescription renewal meets the protocol requirements, the ambulatory care pharmacist could place the order for a prescription renewal without having to ask the PCP. This type of model was not pursued in this study, since the EHHC is a residency-training clinic and many PCPs are resident physicians. Being able to experience managing prescription renewal requests for a panel of patients is an important part of the learning process for family medicine physicians during residency training.

The results of the survey indicate that this activity was beneficial for the education of pharmacy students. For all seven statements, ≥80% of students agreed or strongly agreed. The highest level of agreeance was for the statement that the knowledge and skills learned through this activity can be applied in other pharmacy settings. This is helpful as not all students will end up pursuing a career in ambulatory care, but this activity will still contribute to their education and be applicable to their future settings. The two statements with the lowest level of agreeance were the statements regarding handling drug–drug interactions and regarding confidence in making recommendations to PCPs. Of note, all statements did have a high level of agreeance, but these two had the lowest. Regarding the handling of drug–drug interactions, it is possible that students did not encounter that many situations of drug–drug interactions during this review process. Since these are prescription renewal requests, it would be assumed that the majority of the patients do not have drug–drug interactions that would need intervention, as problematic drug–drug interaction should have been handled during the original prescribing process. Regarding confidence in making recommendations to PCPs, students may still not be fully confident in their clinical knowledge as they are still in their fourth year. Confidence can be improved over time, and it may not be necessary for fourth-year students to have full confidence in making recommendations depending on what part of the APPE year they are.

One large limitation to this study is that it is descriptive in nature and does not include a control group. Since there was no control group, the added benefits of this service cannot be completely quantified compared to the standard of care without this service. There were many recommendations made by pharmacy students and the acceptance rate of the recommendations by PCPs was positive. Although the acceptance rate was high, there is no way to directly assess if these recommendations had any impact on hard outcomes, such as improvement in disease control or decrease in hospitalizations, which makes it difficult to fully assess what type of impact this has on patient care. Although the impact on patient care is not directly observed, it can be concluded that the service has a positive impact on clinic workflow as it can help bring information to the PCPs attention quicker. Anecdotally, this process has received positive comments from providers in the clinic, particularly from the medical residents as they are still in their training. Additionally, it is unknown whether the PCPs would have also been able to identify the recommendations on their own, if the pharmacy student did not send a recommendation. A future study may consider including a control group, to identify if the pharmacy student recommendations were an added benefit, or if the PCPs would have also made the exact same adjustments on their own.

Another limitation is that the assessment of impact of this process on student learning is being assessed in this paper via student-reported responses. Students perceived this activity positively and believe that it contributed to their learning, but this is just the student perception and may not be accurate. One study demonstrated that pharmacy students’ self-evaluations were consistently higher than preceptor evaluations, but did improve throughout the APPE year [6].

This process had a positive impact in the clinic workflow and was reported as highly valuable to the pharmacy students as a learning tool, which is the reason to continue with this process. During APPE rotations, pharmacy students may not receive ample amounts of hands-on experience in prescription order verification. The prescription renewal request review process simulates the idea of prescription order verification, which is a primary responsibility of pharmacists in many settings. This process will continue to be implemented at the EHHC for APPE rotations. Future studies may be conducted to further assess the providers perspective on this service and to assess if this service has any impact on changing prescribing trends.

## 5. Conclusions

APPE pharmacy students were able to make effective recommendations through a prescription renewal request review process. This review process also served as a helpful learning activity for pharmacy students based on student perspective. 

## Figures and Tables

**Table 1:** Acceptance rates of recommendations by category.

Categories	Accepted	Denied (Not Acknowledged)	Denied (Reason Provided)
Total Recommendations (n = 184)	69.57%	16.30%	14.13%
Appointment (n = 23)	69.57%	8.70%	21.74%
Discontinue Medication (n = 8)	50.00%	37.50%	12.50%
Dose Adjustment (n = 7)	42.86%	42.86%	14.29%
Dosage Form Change (n = 10)	70.00%	30.00%	0.00%
Labs (n = 43)	58.14%	25.58%	16.28%
New Medication (n = 2)	50.00%	0.00%	50.00%
Quantity Change (n = 75)	78.67%	9.33%	12.00%
Therapeutic Interchange (n = 16)	81.25%	6.25%	12.50%

**Table 2:** Survey results from student pharmacists after completion of the rotation (n = 35).

Statement (mean)	Strongly Disagree	Disagree	Neutral	Agree	Strongly Agree
The process helped me learn what monitoring parameters are needed for specific drugs (4.37)	0 (0%)	0 (0%)	5 (14.3%)	12 (34.3%)	18 (51.4%)
The process helped me learn how to handle drug–drug interactions (4.17)	0 (0%)	1 (2.9%)	5 (14.3%)	16 (45.71%)	13 (37.14%)
The process helped improved my confidence in my ability to verify prescription orders for safety and efficacy (4.37)	0 (0%)	1 (2.9%)	5 (11.4%)	14 (40%)	16 (45.7%)
The process helped improve my written interprofessional communication skills (4.23)	0 (0%)	2 (5.7%)	5 (14.3%)	11 (31.4%)	17 (48.6%)
The process helped me improve my ability to efficiently navigate the electronic medical record (4.46)	0 (0%)	1 (2.9%)	2 (5.7%)	12 (34.3%)	20 (57.1%)
By the end of the rotation, I felt confident making recommendations to PCPs through this process (4.2)	0 (0%)	1 (2.9%)	3 (8.6%)	19 (54.3%)	12 (34.3%)
I can apply what I learned from this activity to other pharmacy settings in addition to ambulatory care (4.63)	0 (0%)	0 (0%)	2 (5.7%)	9 (25.7%)	24 (68.6%)

## Data Availability

Not applicable.
